# You can’t “nudge” nuggets: An investigation of college late-night dining with behavioral economics interventions

**DOI:** 10.1371/journal.pone.0198162

**Published:** 2018-05-31

**Authors:** Samuel Bevet, Meredith T. Niles, Lizzy Pope

**Affiliations:** 1 Department of Food Systems, University of Vermont, Burlington, Vermont, United States of America; 2 Department of Nutrition and Food Sciences, University of Vermont, Burlington, Vermont, United States of America; University of Tennessee Health Science Center, UNITED STATES

## Abstract

A mixed-methods approach was used to evaluate and improve the “late-night dining” options in a university dining hall. Surveys assessed student desires for late-night offerings, and evaluated students’ habits and motivations during late-night dining. Two interventions were implemented to see if students could be “nudged” into different choice patterns. In the first, a “veggie-heavy” entrée was added at the beginning of the entrée line, so that students would substitute an entrée containing vegetables for the alternatives. In the second, a snack-food bar was set up to cater to students who didn’t want to stand in the long entrée line, and preferred a snack. Data on food choice was collected during the interventions. Survey responses showed significant differences in the reasons females and males utilized late-night dining (p<0.001). We also found that students at late-night dining had a lower emphasis on health than the general student population. Even students at late-night who reported being health-conscious showed no difference in food selections from students who said health was not important (p = 0.883). Veggie-heavy entrées had mild success in increasing vegetable selection. However, veggie-heavy entrées were largely ignored when the other option was chicken nuggets. The snack bar was very popular. Entrée placement and convenience lines may have mild impacts on food selection in a late-night dining environment.

## Introduction

The American College Health Association reports that 33% of college students are overweight or obese [1], and American undergraduates often gain weight while at college [1]. People who become obese or develop poor eating habits during childhood and young adulthood are more likely to struggle with these problems in adulthood [2,3]. This can lead to a variety of illnesses including diabetes, cardiovascular disease, and cancer [4].

Consumption of “junk food” and evening snacks may be large contributing factors in college weight gain [5]. Evidence indicates between 32.5%-72.8% of college students report that they often/always have evening snacks [6,7]. Some research suggests calories consumed late at night contribute to greater weight gain than if the calories were consumed during the day, due to the body’s cyclical metabolism [8]. This means unhealthy late-night meals may be especially detrimental for diet-related health. A study of Korean college students found that living in dormitories was associated with significantly increased calorie intake at night compared to living at home, especially from fried chicken and flour-based foods [9]. However, little research has analyzed late-night eating at American colleges.

Students living in dormitories on American college campuses often consume meals in college dining halls as part of a pre-paid meal plan. These plans often are structured around “all you care to eat” dining experiences where a student can take and eat as much or as little food as they would like each time they enter the dining hall. Dining hall intervention studies have demonstrated the potential for cafeterias to encourage healthy eating choices. These interventions are informed by behavioral economics, which examines the reasons why consumers make decisions in the short-term that may conflict with long-term goals, such as remaining healthy [10]. The positioning of food within both the serving line and cafeteria has been shown to influence the amount and types of food people will take [11–14]. These interventions have been called “Nudging Interventions,” because they subtly push consumers towards healthier choices without removing unhealthy options [10]. While many studies have looked at applying behavioral economics to dining halls, to our knowledge no studies have looked at using these interventions during late-night dining. To address this research gap, the objectives of this study were to examine college students’ perceptions of health and late-night dining, while also implementing two nudging interventions in a late-night dining environment to determine their impact on food choices.

## Methods

Research was conducted at the University of Vermont (UVM), which enrolled 9,786 undergraduate students in the 2016–2017 school year. “Late-night dining” was held Monday-Wednesday from 10:00pm-12:30am at an all-you-care-to-eat cafeteria on campus. Minimal food service staffing during these hours restricted the types of food that could be easily served. Options were usually limited to fried processed foods and dishes that could be premade and quickly reheated, such as chicken nuggets, corn dogs, and pulled pork.

The study was approved by the UVM Committee on Human Research in the Behavioral and Social Sciences and deemed exempt. Participants completing surveys could read a study information sheet before choosing whether or not to complete surveys. The IRB approved our request for a waiver of documentation of consent, as participants’ willingness to complete the survey was considered consent and no identifying information was collected with research data. After IRB approval was received, a survey about late-night dining was administered through a survey delivery program, Campus Labs. The survey was developed collaboratively to collect information of interest to both campus administrators, Dining Services, and researchers. Participants were recruited through an email to all 4,994 students living in on-campus housing. Survey completion was voluntary, and incentivized by the chance to win one of five $25 gift cards towards on-campus dining. Students were asked about their frequency and reasons for attending the university’s late-night dining option. Students used a 7-point Likert scale to rate the importance of health on their late-night food choices (“Health score”), ranging from “Not at all” to “Very important.” Students were also asked about other foods they would like to see offered (if any). Responses were coded into three categories: 1) those wanting healthy options, 2) those wanting less healthy options, or 3) those seeking “more” options [15]. Two researchers (including a Registered Dietitian) coded each response and then compared codes and resolved any coding disagreements by discussing the particular food item and consulting nutrition facts. Requests for fruits, vegetables, lean protein, and whole grains such as a salad bar, fruit bar, or grain bowls were classified as healthy options whereas options high in fat, sugar, or calories such as ice cream, fries, and chicken nuggets were classified as less healthy. Students who requested both healthy and less healthy options or whose requests could not be easily classified as healthy or unhealthy without more information, such as those requesting cereal were classified as wanting “more” options. Table 1 displays survey questions and variables created for analysis.

**Table 1:** Survey questions and variables.

Survey Question	Scale/Options	Variable(s)
How many nights/week do you eat between 10PM and midnight when you are not at UVM?	0–7 nights/week	Home_Habits
How often do you go to late-night dining at Harris-Millis?	Never-3 times/week	Attendance
What time do you plan on going to bed tonight?		Bedtime
Are you satisfied with the options at late-night?	Yes/No	Satisfaction
Are there any particular foods you'd like to see offered at late-night?		Desire_Healthy
		Desire_Unhealthy Desire_More
		Desire_Vegetarian
What is your primary reason for going to late-night at Harris-Millis?	Snack/Meal/Socializing/Bored/Other	Reason_Snack
		Reason_Meal
		Reason_Socialize
		Reason_Bored
How big of a factor is health in your late-night dining choices?	1–7 Likert Scale: 1 = Not at all, 7 = Very Important	Health Score

Following the online survey, a second survey was administered in-person in the cafeteria during late-night dining. This survey contained a subset of the questions from the emailed survey. Additionally, students reported the food they had selected to eat that night. These reported food choices were coded by two researchers as healthy, less healthy, both, or unknown using the same criteria as the Pre-Survey. Due to the anonymous nature of both surveys, it is unknown how many students completed both the Pre-Survey and the At-Late-Night Survey.

Researchers and dining staff worked together to implement two behavioral economics-based interventions at late-night dining during the spring semester of 2017. In the first intervention, vegetable-heavy entrées were added at the beginning of the self-serve line. These entrées were vegetable lasagna (Mondays), broccoli mac-and-cheese (Tuesdays), veggie-egg scramble and a root-vegetable hash (Wednesdays); they were added to the usual options of chicken nuggets (Mondays), pulled pork sandwiches (Tuesdays), and pancakes and sausage (Wednesdays). The intervention options were not necessarily lower in calories and saturated fat or substantially higher in key micronutrients than the traditional options. They were designed to be 1) easy for the small late-night staff to prepare, and 2) appealing enough to late-night diners so that we could test the concept that students could be nudged into taking vegetable-containing entrée options by placing these options at the beginning of a serving line. The intervention was carried out for three weeks, for a total of nine days (late-night dining only occurred on three days out of the week). During this time, researchers stood near the serving line to record the food choices and gender of everyone taking food. Additionally, the number of people coming into the dining hall each night was recorded electronically through the cash register.

In the second intervention, a snack-food convenience line called the “Crunchy Munchy Bar” was added. It was placed beside the salad bar, which Dining Services reported had minimal foot traffic prior to the intervention. Snack foods included chips and salsa, hummus, popcorn, trail mix, yogurt, and pre-cut fruit. The Crunchy Munchy bar was designed to appeal to students who did not want to wait in a long entrée line, and might only be looking for a snack at late night. Researchers tallied how many students took something from the snack food line, broken down by gender. This intervention was also carried out for three weeks, for a total of nine days. During this time, the veggie-heavy entrée intervention was discontinued so that each intervention could be looked at in isolation. Initially, we hoped to assess the effectiveness of both interventions using Dining Services production reports. Unfortunately, these reports were less accurate than we anticipated. Therefore, we did not get the depth of quantitative food production data that would have allowed us to compare our observational data with all food served every night.

Survey responses were analyzed using STATA 15 [16]. Since the majority of the variables were either binary or ordinal, Kendall’s tau correlations were used to determine relationships between variables on the Pre-Survey and At-Late-Night Survey. Statistically significant differences were examined for binary variables using Chi-Square tests. An analysis of variance with Scheffe’s multiple comparison tests [17] was utilized to explore varying outcomes based on different groups and pairwise comparisons among variables with Likert or other numerical outcomes. Finally, to assess the multiple potential variables related to a student’s perceived health score, ordered logistic regressions were used on both the Pre-Survey and the At-Late-Night Survey. Results for the model are reported in odds ratios, which can be interpreted that any coefficient below 1.00 is a reduced odds and anything with a coefficient higher than 1.00 is an increased odds.

## Results

### Surveys

Descriptive statistics for both surveys are reported in Table 2.

**Table 2:** Descriptive statistics.

	Pre-Survey^a^			At-Late-Night		
Variable	N	Mean^b^	S.D.	N	Mean^b^	S.D.
Class Year	647					
First-Year		53.0%				
Sophomore		41.1%				
Junior		3.5%				
Senior		1.2%				
Other		1.1%				
Gender (female = 1)	674 (360)	67.4% (63.1%)				
Home_Habits	626 (369)	2.76 (3.01)	2.03 (2.04)	128	3.40	2.01
Attendance	627					
Never		26.5%				
<1 time/week		42.0%				
1 time/week		12.8%				
2 times/week		11.3%				
3 times/week		5.6%				
Other		2.0%				
Satisfaction (yes = 1)	627 (369)	41.6% (43.1%)		128	72.7%	
Desires	409 (268)					
Desire_Healthy		39.6% (36.6%)				
Desire_Unhealthy		30.1% (33.6%)				
Desire_More		29.8% (29.5%)				
Desire_Vegetarian (yes = 1)	409 (268)	10.8% (11.6%)				
Reasons	482 (369)			141		
Reason_Snack		35.9% (34.7%)			21.3%	
Reason_Meal		33.5% (31.7%)			44.7%	
Reason_Socialize		24.3% (25.7%)			24.8%	
Reason_Bored		2.3% (2.4%)			5.0%	
Reason_Other		4.1%			4.3%	
Health (1–7 scale)	627 (369)	3.94 (3.75)	1.92 (1.88)	127	3.48	1.94

*Note*. ^a^Values in parentheses denote the subset of respondents who reported attending late-night *at least* once per week.

^b^Categorical variables reported as percentages.

#### Pre-survey

Our Pre-Survey received 681 responses for a response rate of 13.6%. Students provided open-ended answers to the question “Are there any particular foods you would like to see offered at Late-Night?” Of these responses (N = 409), 39.6% of students requested only healthier options, 30.1% of students requested only less healthy options, and 29.8% requested a combination of both. Additionally, 10.8% of all these responses made explicit requests for more vegetarian/vegan options. The mean Health Score for respondents was 3.94, with 14% of respondents reporting a Health Score of 1 meaning that health was not at all a factor in their late-night food choices. An ANOVA test compared Student Health Scores between those that reported attending late-night at least weekly (mean Health Score = 3.75, *SD* = 1.88) and those who reported going less than weekly/never (mean Health Score = 4.23, *SD* = 1.94), which was statistically significant difference between the groups (*F* = 9.68, *p*<0.01).

ANOVA results also examined whether gender was correlated with different reasons for attending late night. Overall, 49% of males compared to 25% of females reported attending late night for a meal, a statistically significant difference (Chi2 = 53.89, *p*<0.001). Conversely, fifteen percent of males compared to 33% of females reported attending late-night for socializing (Chi2 = 4.91, *p*<0.001). There was no significant difference in gender for those attending late night for a snack or because they were bored. Additionally, ANOVA results examined the relationship between variables (Desires), (Satisfaction), and (Health Score). Students that exclusively wanted less healthy options (Desire_Unhealthy) were significantly more likely to have a lower Health Score (*M* = 2.66, *SD* = 1.57) than other students (*M* = 4.72, *SD* = 1.77, *F* = 124.28, *p*<0.001). Fifty-seven percent of students who wanted less healthy options (Desire_Unhealthy) were satisfied with the food offered at late-night dining, compared to 21% for other students, which was statistically significant (Chi2 = 52.12, *p*<0.001). Conversely, students who requested healthier options (Desire_Healthy) had significantly higher Health Scores (*M* = 5.35, *SD* = 1.60) than students who requested other types of food (*M* = 3.28, *SD* = 1.73, *F* = 147.67, *p*<0.001). Only 19.7% of students who requested healthy options (Desire_Healthy) were satisfied with the food at late-night dining, compared to 40.5% of other students, which was significantly different (Chi2 = 19.24, *p*<0.001).

#### Pre-survey model

A logistic regression model was run to look at factors influencing a student’s Health Score (Table 3). We found that students who more frequently ate late-night meals when home from college (Home_Habits), students who were more satisfied with the current late-night offerings (Satisfaction), and students who attended late-night dining primarily for snacking or socializing (Reason_Snack; Reason_Socialize), all had significantly greater odds of having a lower Health Score *(p*<0.05). Students who desired healthy options (Desire_Healthy) and students who desired vegetarian options (Desire_Vegetarian) both had increased odds of having a higher Health Score, but only Desire_Healthy was statistically significant (*p*<0.01).

**Table 3:** Ordered logistic regressions for student health scores.

	Pre-Survey^a^			At-Late-Night Survey^b^		
Variable	Odds Ratio	Std. Err.	P>|z|	Odds Ratio	Std. Err.	P>|z|
Home_Habits	0.8693626	0.0415414	0.003	0.9921917	0.0916186	.932
Attendance	0.8790754	0.0641382	0.077			
Satisfaction	0.5502334	0.1135728	0.004	0.1987938	0.0858332	<0.001
Bedtime				0.9635462	0.1653251	0.829
Desire_Healthy	4.402831	1.048484	<0.001			
Desire_Unhealthy	0.3459277	0.0870566	<0.001			
Desire_Vegetarian	1.305367	0.4030509	0.388			
Reason_Snack	0.5849335	0.1547966	0.043	2.249482	1.319639	0.167
Reason_Meal	0.6100174	0.1769659	0.088	2.979909	1.709523	0.057
Reason_Socialize	0.4325845	0.1234397	0.003	1.868892	0.9909147	0.238
Reason_Bored	0.5260575	0.3570424	0.344	4.374742	3.668882	0.078

*Notes*. ^a^N = 409, Pseudo R2 = 0.1217.

^b^N = 118, Pseudo R2 = 0.0554.

#### At-late-night survey

One hundred and twenty eight students agreed to take the in-person survey conducted during late-night dining. Descriptive statistics are reported in Table 2. Although we did not explicitly ask for class year on this survey, the majority of respondents were likely to be freshman and sophomores, as they almost exclusively are the students who live on campus and would be attending late-night dining. The mean Health Score of students surveyed at late-night dining was 3.48. Notably, 24% of respondents at late-night dining chose a Health Score of 1, indicating health was “not at all” a factor in their late-night dining choices (as compared to 14% in the Pre-Survey).

ANOVA tests were used to compare each student’s Health Score to their actual reported food choices. There was no statistically significant difference between a student’s Health Score and the foods they actually took at late-night dining, (*F =* 0.39, *p* = 0.883), indicating that how important health was for their late-night dining options did not relate to actual entrée choice.

A logistic regression model was run to look at factors influencing a student’s Health Score at late-night dining (Table 3). Students who reported being unsatisfied with the offerings at late-night dining (Satisfaction) had significantly increased odds of having a higher Health Score (*p*<0.001). No other factors from the At-Late-Night Survey had any significant influence on a student’s Health Score.

### Nudging interventions

**Fig 1:**
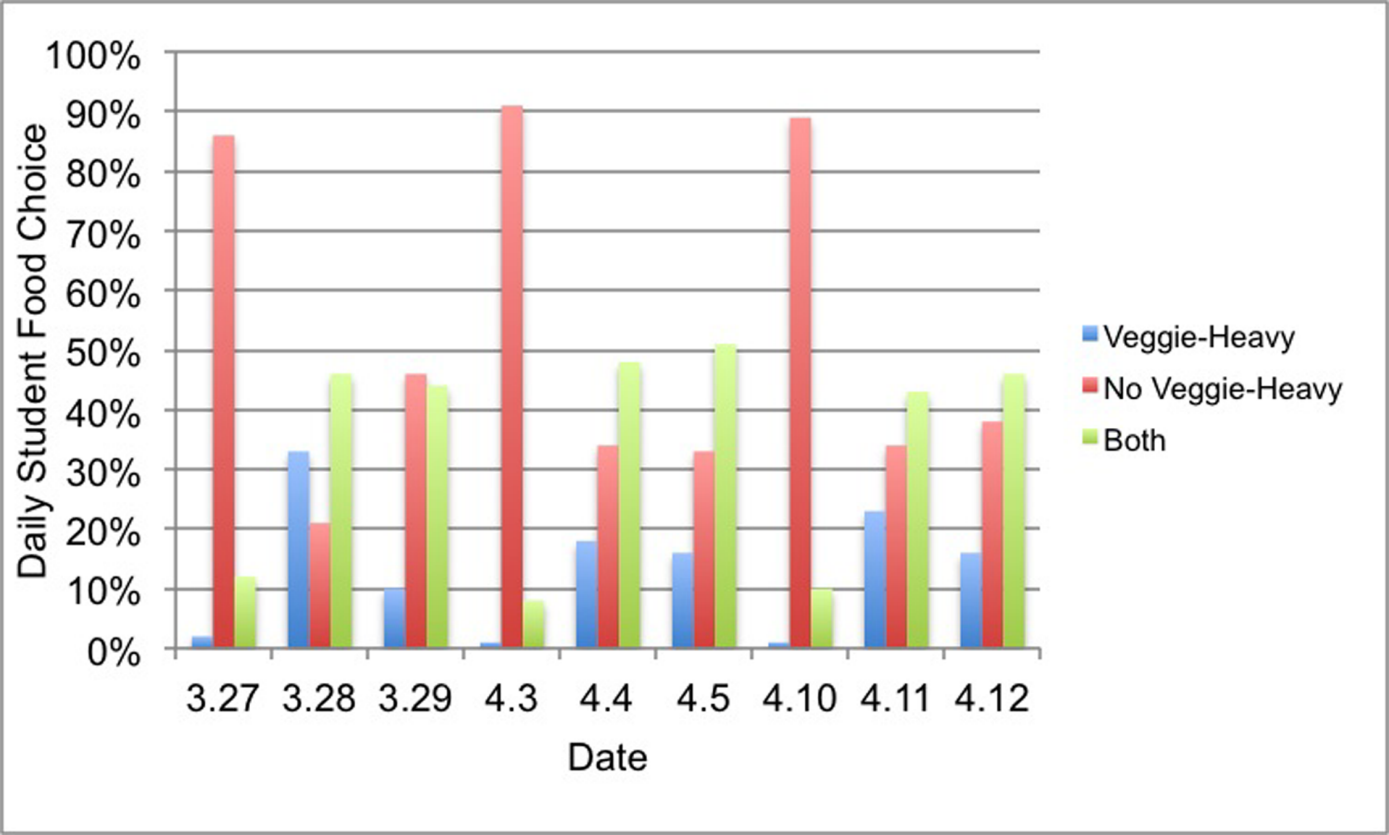
Vegetable-heavy intervention observations. Percentage breakdowns of daily student food choice during our first intervention period.

Vegetable-heavy entrées were added to the main entrée line for three weeks, from March 27-April 12. During this time, researchers observed 2,397 trips through the entrée line, 28% by females and 72% by males. Student food choices during the vegetable-heavy entrée intervention period are shown in Fig 1. March 27, April 3, and April 10 are all Mondays when chicken nuggets were served alongside vegetable lasagna. A sharp contrast between those three Mondays and the other six dates can be seen. On Tuesdays and Wednesdays, between 54%-79% of students incorporated a vegetable-heavy entrée into their late-night dining selection (Veggie-Heavy + Both). On chicken nugget Mondays, only between 9%-14% of students took a serving of the vegetable lasagna.

After the vegetable-heavy entrée intervention concluded, the Crunchy Munchy healthy snack food bar was implemented. Over the course of three weeks, students made 1,975 trips to the snack bar. Gender breakdown for usage was 51% female, 49% male. Qualitative assessment of student feedback (shown through quotes in Table 4), suggest that the Crunchy Munchy Bar led to increased selection of (and excitement about) healthy foods.

**Table 4:** Examples of student feedback.

A Selection of Student Food Requests From the Pre-Survey	Student Quotes at Crunchy Munchy
TASTY GLUTEN FREE DRUNK FOOD gluten free mac and cheese, gluten free pizza, etc.	I just ate mushrooms at late night, it was an incredible experience.
Healthy ones, normal people food like not corn dogs and fries ew	There’s raspberries!
Have the corn dogs more often	This is awesome
At night I am looking for snacks. The late-night dining choices… almost encourage eating an entire full meal, and that’s often what happens as a result even though it is unnecessary.	Actually, like, decent food
I like the options, love the chicken nuggets	They have crunchy munchies!
Something warm. To help sleep. like soup.	

## Discussion

From our two surveys, we gain some important insight into the way college students think about late-night food and health. We find key differences between the responses of students completing the Pre-Survey away from late-night dining compared with students taking the At-Late-Night Survey while in the late-night environment. We also see no correlation between students’ health goals and their behaviors during late-night dining.

The mean student Health Score on the Pre-Survey was 3.94, and 3.75 for students who reported attending late-night dining at least once per week, while the mean Health Score of students at late-night dining was 3.48. While we cannot compare these statistically since the samples are not the same, this suggests that in the moment while at late-night dining, students place a lower emphasis on healthy eating than they do at other times. This could be due to projection bias, which is when a person incorrectly estimates how they will react in a future situation [18]. Students taking the Pre-Survey in a “cold” logical state imagine that health will be a very important factor in their future food choices; this ends up not being true when they arrive at late-night dining in a “hot” visceral state [19]. When not at late-night dining, students overestimate the importance of health on their decision-making. After a long day of classes and homework, students place less emphasis on health and are more interested in fulfilling their immediate cravings for comfort food.

Student food choices during late-night dining were not significantly impacted by student Health Scores. Students were just as likely to choose vegetable-heavy options if they said health was “very important” or “not at all important” to their food choices at late-night dining. Students may not have perceived the vegetable-heavy entrées to be that much healthier than the original options. Alternatively, this could also be due to what O’ Donoghue & Rabin [20] refer to as *present-biased preferences*: students put more weight on their immediate preferences (eating chicken nuggets) than their long-term goals (eating more vegetables). The tendency to make decisions in the present that are immediately rewarding versus making decisions that might lead to greater gains in the long term can also be explained by hyperbolic discounting theory. Hyperbolic discounting refers to the tendency for consumers to pick smaller-sooner rewards rather than larger-later rewards that they would need to wait for, especially if the smaller-sooner reward is immediately available [21]. In this study, students in the late-night dining environment seemed more likely to indulge their hedonic or taste preferences rather than make choices that might be consistent with their long-term health goals, effectively discounting the importance of the health goals. This can present a challenge for dining services trying to satisfy student desires. In “cold” states, students request that healthier options be offered; however, when they are in “hot” states they walk right past the healthy options and head for the junk food. This disconnect between students’ stated desires and actions can be frustrating for dining service administrators, and may encourage the dining service to simply continue catering to students’ “hot” states.

Students’ present-biased preferences could potentially be exacerbated by intoxicants such as alcohol or marijuana. As Ajzen [22] notes, “performance of a behavior is a joint function of intentions and perceived behavioral control” ([22], p. 185). While students may have the intention of eating healthfully, intoxicants may reduce their ability to regulate their own behaviors. We did not collect data on rates of student intoxication, but multiple students were overheard saying they were currently under the influence of marijuana, and another study identified alcohol as an influencing factor in students’ late-night food consumption [23]. Future research on the role intoxicants play in student food choice is needed.

We observed an interesting gender divide between the entrée station and the Crunchy Munchy bar. Only 28% of students using the entrée line were female, while 51% of Crunchy Munchy bar trips were made by females. This is consistent with our survey responses, where women reported being much more likely to attend late-night dining for a snack or to socialize, while men were much more likely to go for a meal. Counihan [24] attributes gendered eating differences in America to cultural food norms, where men are socially encouraged to consume large amounts of hearty food while women are encouraged to more sparingly eat healthy items. However, Wichianson, Bughi, Unger, Spruijt-Metz, & Nguyen-Rodriquez [25] identified stress as a common reason for college students’ nighttime food consumption, and found that among their sample, men were more likely than women to use maladaptive eating practices to try to manage stress. Understanding the gender division in late-night dining would be beneficial both for student health and college dining services.

Our nudging interventions appear to have been partially successful in increasing the choice of vegetable-heavy entrées and snack foods. Based on our observational data, we know that students were incorporating more vegetables into their diet than they otherwise would have, since prior to the intervention no vegetables were served in the late-night entrées. However, one unintended consequence of these interventions may have been that the additions of new vegetable and snack options just led to more food selection, rather than a reduction in less healthy selections. It is likely that if students took snacks from the Crunchy Munchy Bar and a late-night entrée they would be eating additional food. Similarly, taking a large portion of snacks, even healthy ones, from the Crunchy Munchy Bar may result in the same health/weight outcomes as just eating the original entrées. Increased consumption of vegetables and fruits is often suggested as a way to promote healthy weight due to their low energy-density and high fiber content [26]. However, Djuric et al. [27] observed a six-pound weight gain among women who only focused on increasing vegetable and fruit consumption without also focusing on reducing fat intake. Another study found that some vegetables were associated with weight loss, while others were associated with weight gain [28]. Solely emphasizing vegetable and fruit consumption may not be enough to positively influence college student health in dining halls. We would need more concrete data on how much food was served to draw conclusions about the interventions’ effectiveness at improving student health.

Chicken nugget Mondays appear to have been mostly impervious to nudging interventions. Although we did see a drop in nugget servings per student, around 90% of students on Mondays ignored the vegetable lasagna in favor of the chicken nuggets and french-fries. Several factors might be in play here. The first is that self-serving the lasagna from the tray took a bit more effort than scooping up nuggets. The lasagna was pre-cut, but students had to use a spatula to separate and serve pieces. This could slow down their progression, while dozens of other hungry college students waited behind them. Research has shown that even mild increases in the effort needed to access food can reduce selection [29]. The second factor is that students may just have a strong preference for chicken nuggets, or a strong preference against vegetable lasagna, that cannot be overridden by nudging interventions. One study targeting elementary students tried to increase fruit consumption over french-fry consumption by making apple slices the default item served, but given the option, 96% of students switched their apple slices for fries [30]. As long as french-fries were available, students took them. Similarly, when chicken nuggets were offered, students were able to ignore the nudging intervention, skipping right over the vegetable lasagna and loading up their plates with piles of nuggets. Finally, there could have been a “Monday effect” for chicken nugget choice where the stress of starting a new week and anticipating the week ahead led to more students choosing chicken nuggets and being impervious to the nudging intervention. These results demonstrate some potential limitations for nudging interventions to positively influence consumer health.

Our interventions concluded at the end of the spring semester. The following fall semester, dining services continued to offer both vegetable-heavy entrées and the Crunchy Munchy bar. Instead of using the nudging intervention, vegetable-heavy entrées are the only option served on some nights. The campus head chef reported that this may potentially reduce food costs, because students were previously over-serving themselves the less healthy options. He also noted that the Crunchy Munchy bar has continued to be popular with students. Dining services also decided to only serve chicken nuggets occasionally, rather than every Monday, to make nugget night less of a habitual weekly event.

This research had several limiting factors. Our Pre-Survey and At-Late-Night Survey had population overlap; therefore while we were able to note differences between the two groups, we were unable to compare them statistically. Students were asked about the role health plays in their decision-making, but “health” was self-defined by each student. We focus on physical health here, but students may have considered mental health in their answers. To gain a greater understanding of how student preferences change between “hot” and “cold” states, future research should include more nuanced definitions of health, and either track the same student responses over time or ensure statistically independent samples. Our conclusions were also limited by problems with the Dining Services production report data, which made it difficult to assess our interventions’ effects on less healthy food selection. More robust tracking of the food served and wasted by students would be beneficial for evaluating cafeteria nudges. The vegetable-heavy entrées we chose to offer may still have been high in fat and calories, and therefore not seen as “healthy” by students at late-night dining. A nutrition analysis of generic versions of each entrée indicated that the veggie-heavy entrées were lower in fat, sugar, and sodium as well as higher in Vit A and Vit C than generic versions of the normal entrées, but similar in calories and saturated fat. Using entrées that are lower in fat and calories may better illustrate whether late-night dining food choice is associated with overall interest in one’s health. Finally, our study was limited to repeated menu offerings of the same foods on the same days each week. Randomized control trials evaluating different food pairings on different days of the week, and comparison treatments using other non-vegetable entrees, could help control for additional factors influencing student food choice. This would allow for better evaluation of the role food-positioning plays in food-selection. The biggest strength of our research was taking a mixed-methods approach to investigate a relatively unstudied area of student dining. Through a mixture of quantitative, qualitative, and observational data, we were able to create a baseline understanding of students’ late-night dining behavior that can inform future research.

## Conclusion

From our survey data, we concluded that the stated importance of health on food selection did not have a relationship to actual student food choice. We also found that, on average, students not at late-night dining placed a higher value on health than students attending late-night dining. We found a significant difference in the reasons males and females attended late-night dining, with males more likely to go for a meal and females more likely to go to socialize. Although we do not know whether our nudging interventions decreased less healthy food selection, they were effective at increasing vegetable selection in at least some contexts. The exception to this was during chicken nugget nights, where students demonstrated their overwhelming preference for nuggets. For colleges and dining services looking to positively impact student health, it is important to assess the strengths, but also the limitations, of nudging interventions within the dining hall.

## Supporting information

S1 Dataset Pre-survey anonymous—This file contains the data from the survey conducted when participants were not at late-night dining. (XLSX)

S2 Dataset At-late-night survey data—This file contains the data from the survey conducted at late-night dining. (XLSX)

S3 Dataset Late night dining survey data—This file contains data about participants choice of entrée during the veggie-heavy entrée first in line intervention. (XLSX)

S4 Dataset Crunchy Munchy comparison data—This file contains data about foodx choice during the Crunchy Munchy Bar intervention. (XLSX)
